# DBAC: A simple prediction method for protein binding hot spots based on burial levels and deeply buried atomic contacts

**DOI:** 10.1186/1752-0509-5-S1-S5

**Published:** 2011-06-20

**Authors:** Zhenhua Li, Limsoon Wong, Jinyan Li

**Affiliations:** 1 Bioinformatics Research Center, School of Computer Engineering, Nanyang Technological University, Singapore 639798; 2 School of Computing, National University of Singapore, Singapore 117417

## Abstract

**Background:**

A protein binding hot spot is a cluster of residues in the interface that are energetically important for the binding of the protein with its interaction partner. Identifying protein binding hot spots can give useful information to protein engineering and drug design, and can also deepen our understanding of protein-protein interaction. These residues are usually buried inside the interface with very low solvent accessible surface area (SASA). Thus SASA is widely used as an outstanding feature in hot spot prediction by many computational methods. However, SASA is not capable of distinguishing slightly buried residues, of which most are non hot spots, and deeply buried ones that are usually inside a hot spot.

**Results:**

We propose a new descriptor called “burial level” for characterizing residues, atoms and atomic contacts. Specifically, burial level captures the depth the residues are buried. We identify different kinds of deeply buried atomic contacts (DBAC) at different burial levels that are directly broken in alanine substitution. We use their numbers as input for SVM to classify between hot spot or non hot spot residues. We achieve F measure of 0.6237 under the leave-one-out cross-validation on a data set containing 258 mutations. This performance is better than other computational methods.

**Conclusions:**

Our results show that hot spot residues tend to be deeply buried in the interface, not just having a low SASA value. This indicates that a high burial level is not only a necessary but also a more sufficient condition than a low SASA for a residue to be a hot spot residue. We find that those deeply buried atoms become increasingly more important when their burial levels rise up. This work also confirms the contribution of deeply buried interfacial atomic contacts to the energy of protein binding hot spot.

## Background

Protein-protein interactions are dominated by hydrogen bonds, salt bridges and hydrophobic contacts across the interface [1,2]. These local interactions have to be desolvated, densely packed, and hence deeply buried to make contribution to the binding free energy [3-6]. This is why the energetically important hot spot residues in the interface tend to cluster into local regions with low solvent accessible surface area (SASA) values [7,8].

Identifying these energetically important residues, which can offer useful information to protein engineering and better understanding of protein-protein interaction [9], is usually done by site-directed alanine mutagenesis. This experimental method mutates the target residue into alanine which only has a C*^β^* heavy atom on its side-chain [10,11]. A residue whose mutation results in a large binding free energy change (≥2.0 kcal/mol, for example) is defined as a hot spot residue [12].

Many feature-based [13-17] energy-based [18-23] and even feature-and-energy-combined [24,25] computational approaches have been proposed to address the hot spot prediction problem. Almost all of these feature-based methods use SASA information of the residue as a critical feature in the prediction. A low SASA is necessary for a residue to be a hot spot residue; however, it is not sufficient, as a large number of non hot spot residues also have low SASA values. Therefore, SASA is not effective for distinguishing between slightly buried residues—a large part of which are non hot spot residues—and deeply buried residues that are very likely to be hot spot residues.

In this work, we introduce a new descriptor for protein atoms and residues. It is named “burial level”. In the definition of burial level, the buried immobilized water molecules are treated as an integral part of the protein complex. We show that our definition of residue burial level is nicely correlated to ∆∆*G*. A high burial level is not only in general necessary for hot spot residues but also more sufficient for them in comparison to SASA. In other words, most hot spot residues tend to have high burial level while most non hot spot residues are exposed or just slightly buried. We also define the burial level of atomic contacts and we determine the number of three types of buried interfacial atomic contacts at different burial level that are directly broken when the residue is substituted by alanine. The number of those deeply buried atomic contacts together with the burial level of the residue itself are further fed into SVM as features to classify interfacial residues into hot spot residues or non hot spot residues. We name this SVM-based model DBAC since the features used are mainly based on the Deeply Buried Atomic Contacts. By applying our method to a data set of 258 mutations, we achieve an F measure of 0.6237 under the hot spot definition of ∆∆*G* ≥ 2.0 kcal/mol, which is better than other computational methods. We also conduct a detailed analysis of the features used in this work; and we find that hot spot residues tend to have significantly more deeply buried atomic contacts than non hot spot residues.

## Methods

### Data set

Our data set is collected by retrieving the experimental alanine mutagenesis data from the alanine scanning energetics database (ASEdb) [26] and other previously published works [27-31]. We require that: the 3D structure of the wild-type protein complex is solved by X-ray crystallography and is reported in PDB [32], and the associated solvent information is also included in the PDB file. We do not consider protein-ligand interaction or protein-peptide interaction in this work; thus those interactions without an extended interface are excluded. The reason is that the interfaces of protein-ligand interactions are small and most interfacial residues are exposed in the solvent to a certain degree; thus the burial levels of the atoms are mostly very low and imply little information. The structural similarity of the complexes are tested by the CE algorithm [33]. If the two chains of the two complexes have a significant similarity, their binding interfaces are further examined to ensure that there is no redundancy in the data set. Furthermore, only mutations in the interface are considered. We used another version of this data set in our previous work [34], where the requirement that the mutations have to be in the interface was not applied.

Our data set in this work consists of 258 mutations distributed in 13 protein complexes. Hot spot residues are usually defined by setting ∆∆*G* ≥ 1.0 kcal/mol or ∆∆*G* ≥ 2.0 kcal/mol. We prefer the second choice, as only a higher ∆∆*G* threshold can reflect the direct influence of the mutation. That is, the interfacial atomic contacts that are directly broken by the mutation are taken into consideration with more weights. Under the ∆∆*G* ≥ 2.0 threshold, there are 50 hot spot residues and 208 non hot spot residues in our data set. Some researchers even suggested that a residue should have a ∆∆*G* higher than 4.0 kcal/mol so as to have a strong impact on the binding of the two proteins [9]. In practice, a lower value has to be considered in order to get enough data for statistical analysis [9].

The data set is available at http://155.69.2.25:8080/DBAC data.

### Feature generation

#### Burial level for an atom, residue or an atomic contact

Our definition of burial level is based on atomic contact graph. The atomic contact graph of a protein complex is an undirected graph with heavy atoms as nodes and atomic contacts as edges. The atoms in this graph are labeled as exposed or buried according to its SASA. If the SASA of an atom is not less than 10.0Å ^2^, it is exposed, otherwise it is buried. SASA is calculated by the NACCESS software based on the Lee-Richards algorithm [35]. All the *exposed* water molecules, which we consider as a part of the bulk solvent, are removed, while the buried water molecules are kept as a part of the complex. Thus the oxygen atoms of the buried water molecules are a part of the atomic contact graph.

The atomic contact is defined by a distance threshold and the Voronoi diagram. Voronoi diagram decomposes the 3D space into cells by assigning every point in the space to its nearest neighboring input site. Here in this work, the input sites are the positions of the atoms in the complex structure. If two atoms’ Voronoi cells are adjacent to each other, they are somehow “sheltering” each other. We define the atomic contact by adding an extra distance requirement to Voronoi diagram. Two atoms are considered to be in contact if they have a distance less than their Van der Waals radius plus the diameter of a water molecule (2.75 Å) and they share a Voronoi facet. We actually used the Delaunay diagram that is dual to Voronoi diagram. Two facet-sharing Voronoi cells correspond to two connected points in the Delaunay diagram. The Delaunay diagram is calculated by using the qdelaunay program in qhull [36]. This distance threshold, 2.75 Å, is based on a water-free idea and it has been used in [37].

In an atomic contact graph, the burial level of an atom is defined as the length of the shortest path from this atom to its nearest exposed atom. For example, the burial level of exposed atoms is 0 and the burial level of their immediate buried neighbors is 1. We calculate the burial levels by adding a pseudo node, which represents bulk solvent, to the atomic contact graph. This node is connected to all of the exposed nodes directly. Then the burial level of any atom equals to the length of the shortest path from this atom to the pseudo node minus 1. This is exactly the single-source-shortest-path problem and it can be solved using Dijkstra’s algorithm [38].

The reason for using a Voronoi-diagram-combined definition of atomic contact is as follows. If only distance information is used, there will be many false atomic contacts in the atomic contact graph whose two atoms cannot contact with each other at all (as they do not share Voronoi facet thus they are spatially blocked by other atoms), and the atomic contact graph will be a trivial discretization of the Euclidean distance between atoms, and the atom burial level will only depend on the distance of the atom to the surface of the complex, especially when a large distance threshold (2.75 Å) is used. Adding Voronoi diagram to the definition makes the burial level depend also on the organization of the atoms inside. Intuitively, the burial level of atoms in a protein complex depends on the size of the protein complex. In general, the larger the protein complex is, the more deeply buried atoms there are. Burial level also depends on the shape of the interacting proteins. For example, globular proteins and protein complexes generally have more deeply buried atomic contacts than those with other shapes.

Note that the calculation of burial level requires information on buried water molecules. In our previous work [34], we have systematically analyzed the contribution of water molecules to the calculation of burial level as well as to protein binding hot spots.

**Figure 1:**
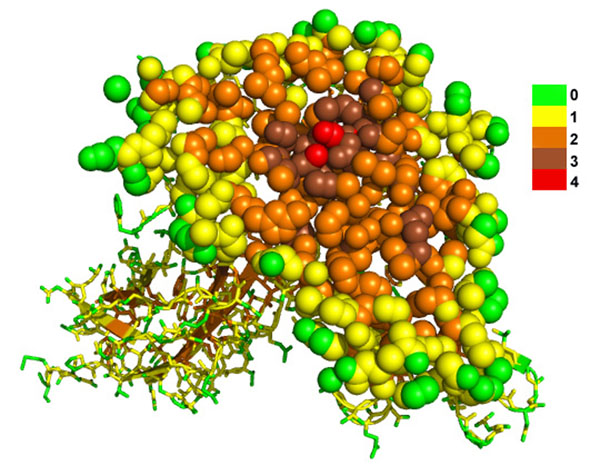
**Cross-section of a growth hormone and growth hormone receptor complex** Cross-section of a growth hormone and growth hormone receptor complex [PDB:1A22] showing the burial level pattern. A layer of atoms in the front are shown as spheres while those at the back are shown as sticks. The colors from green to red indicate the burial level of atoms from 0 to 4. The largest burial level is 4 here. The largest burial level of atom within a protein/protein complex depends on its size, shape and the nearby atoms’ organization.

Figure 1 shows a burial level pattern inside a growth hormone and growth hormone receptor complex. As seen from the figure, atom burial level is indeed a good indicator to describe the extent to which an atom is buried inside a protein or a protein complex. It is clear that the burial level of any two neighboring atoms can have a difference of at most 1. Because the complex is not perfectly globular, burial level 2 is “thicker” with more atoms.

The burial level of a residue is the average value of the burial levels of all atoms in the residue. For an atomic contact, if the burial levels of the two atoms are the same, the burial level of the atomic contact is taken as the burial level of the two atoms, otherwise it is defined as the smaller one of the two burial levels. The difference of the burial levels of two contacting atoms is at most one.

There are some existing concepts that are related to burial level. In [39-43], the authors defined their concept of depth of an atom as the Euclidean distance to the closest exposed atom or to the closest surface water molecule. There are even some sequence-based methods [44-46] that are capable of predicting its value. This definition is only based on the Euclidean distance and hence it cannot capture the contacts between atoms or the organization of the atoms. And the calculation of the shortest distance contains an exhaustive search among all exposed atoms or surface water molecules for every buried atom, while our graph-based concept of burial level can be easily calculated by transferring the calculation into a single-source-shortest-path problem.

#### Directly broken atomic contact, atomic contact types and features

When a residue is mutated into alanine, some interfacial atomic contact are directly broken because of the removal of certain atoms; Some other interfacial atomic contacts may also be broken or distorted due to the conformational change in the local region [31]. Given a residue, we consider its directly broken interfacial atomic contacts, namely those contacts formed by an atom other than *C*, *N*, *O*, *C^α^* or *C* (these atoms are the remainder after a residue is mutated into alanine) and any contact partner from the other chain, even its backbone atoms. For example, for a serine in a complex, the atomic contact between its O^γ^ and a C*^α^* of any residue from the interacting partner is a directly broken atomic contact of this serine, while the contact between its C*^β^* and whatever atom from the interacting partner is not. We classify the atomic contacts into three types. If a contact is between a positively charged atom and a negatively charged atom, which usually corresponds to a salt bridge, it is called a Type-I contact. If a contact is between a hydrogen bond donor and a hydrogen bond acceptor, which usually is a hydrogen bond, it is classified as Type-II contact. Contacts that are neither Type-I nor Type-II are classified as Type-III. Here, the definitions for positively charged atoms, negatively charged atoms, hydrogen bond donors and hydrogen bond acceptors are as given in [8]. We do not further divide the Type-III contacts into subtypes such as other polar contact, hydrophobic contact and so on because they are all not as specific as Type-I and -II contacts. Note that the definitions for Type-I and Type-II contacts are not exactly the same as salt bridges and hydrogen bonds in terms of geometrical requirements, yet they can be still very important [47].

In this work, we use deeply buried atomic contacts whose burial level is not less than 2. We refer to atomic contacts at burial level 0 as exposed atomic contacts and those at burial level 1 as slightly buried atomic contacts. Let *C*(*i*, *j*) denote the number of Type-*i* directly-broken interfacial atomic contacts at burial level *j* of a residue. Then our model uses 6 features to describe a residue: *C*(*I*, ≥ 2), *C*(*II*, 2), *C*(*II*, ≥ 3), *C*(*III*, 2), *C*(*III*, ≥ 3), plus the burial level of the residue. An SVM model based on this feature set is named DBAC (**D**eeply **B**uried **A**tomic **C**ontacts). For comparison, we have also built another model named AC (**A**tomic **C**ontacts) based on another feature set comprising *C*(*I*, 0), *C*(*I*, 1), *C*(*I*, ≥ 2), *C*(*II*, 0), *C*(*II*, 1), *C*(*II*, 2), *C*(*II*, ≥ 3), *C*(*III*, 0), *C*(*III*, 1), *C*(*III*, 2), *C*(*III*, ≥ 3), and the burial level of the residue. The maximum value of burial level depends on the size of the protein complexes, the size of the interfaces as well as the shape of the complex and the organization of the atoms. In general, very few contacts have burial level larger than 3, so we do not distinguish further burial levels larger than 3. For Type-I contact, there are very few cases that have burial level larger than 2, thus we do not use *C*(*I*, > 3) as a feature but merge it with *C*(*I*, 2) into *C*(*I*, ≥ 2).

### SVM training-testing protocol

Support Vector Machines (SVMs) are widely used in many classification and regression problems. They have also been adopted in hot spots prediction problems [15,17,24] with various feature sets and training-testing protocols. In this work, we use the LIBSVM software [48], which is a tool for SVM model training and testing available at http://www.csie.ntu.edu.tw/~cjlin/libsvm. We use the radial basis function (RBF) as the kernel. We do not conduct feature selection because our method is straightforward, and the number of features is not large. However, we evaluate performance on two different feature sets: the deeply buried atomic contacts only (by DBAC) and all the atomic contacts (by AC). The latter feature set is evaluated just for comparison.

The performance is evaluated under leave-one-out cross-validation. To avoid over fitting, we have strictly followed a nested-loop cross-validation procedure. There are 258 mutations in our data set, each time one mutation is taken as the test data and the remaining 257 mutations are used to train the model. The two parameters, namely cost and gamma, are optimized on the training data by a grid search. The grid search evaluates the performance, F measure, of SVMs with different parameter values on the training data using 5-fold cross-validation, and the parameter values with the best performance are chosen to build a training model on the training data. This training model is then applied to the test data, that is, the mutation held out in advance. This procedure is repeated 258 times until every mutation in the data set is tested.

### Metrics in performance evaluation and statistical analysis

Performance is measured by sensitivity, precision, specificity, accuracy and F measure (F1). These measures are defined asfollows: , , , , and  , where TP, FP, TN and FN are the number of true positives, false positives, true negatives and false negatives, respectively. A better classifier should predict hot spot residues with less false positives and less false negatives; thus the F measure, which combines sensitivity and specificity, is used to indicate the overall performance.

We also test the significance of the difference in ∆∆*G* values of predicted hot spot and non hot spot residues. A classifier divides the mutations in the data set into two groups: computational hot spot residues and computational non hot spot residues. The significance of the ∆∆*G* value difference in these two groups are tested by Mann-Whitney test [49]. The result of a classifier with higher F1 value can be less significant when its false positives have very low ∆∆*G* values (near 0 kcal/mol or even negative) and the false negatives have high ∆∆*G* values.

We also examine the value distribution of individual features in hot spot and non hot spot residues. The significance of the difference in the two classes is also tested by Mann-Whitney test.

## Results and Discussion

### Performance of hot spot residue prediction

As introduced, 5 new features are derived from those deeply buried interfacial atomic contacts which are directly broken by alanine substitution. The feature values of a residue are then fed into SVM together with the overall residue burial level to predict whether this residue is a hot spot residue or not. The performance under leave-one-out cross-validation is shown in the second row of Table 1. We achieve an F measure of 0.6237, when ∆∆*G* ≥ 2.0 is used as the threshold to define hot spot residues. The precision of our method is higher than the recall, which means that there are fewer false positives than false negatives. A reason for this is that our model emphasizes the contribution of directly broken atomic contacts. The contacts that are broken or newly formed by the conformational change during the mutation are hard to define quantitatively. The ∆∆*G* of some hot spot residues, whose mutation results in a large conformational change, cannot be fully explained by its directly broken atomic contacts. This is reflected in the lower sensitivity value. The non hot spot residues, whose ∆∆*G* is low, tend to have fewer deeply buried directly broken atomic contacts, leading to a smaller number of false positives and hence a higher precision. We have also evaluated the performance of using the AC feature set containing 12 features, which take exposed and slightly buried atomic contacts into consideration as well. As shown in the third row of Table 1, the performance is not improved even though extra exposed and slightly buried atomic contacts are added to the feature set. Rather, the F measure is driven down to 0.4 and the statistical significance is reduced a lot as well. The reason is presented later.

**Table 1:** Performance of our method (DBAC) in comparison with using all atomic contact (AC) and Robetta

Method	Sensitivity	Precision	Specificity	Accuracy	F1	p-value
DBAC	0.58	0.6744	0.9327	0.8643	0.6237	3.0280×10^–12^
AC	0.32	0.5333	0.9327	0.8140	0.4	1.2849×10^–5^
Robetta	0.44	0.3667	0.8173	0.7442	0.4	5.3817×10^–8^
FoldX	0.5	0.4630	0.8606	0.7907	0.4808	6.2451×10^–11^

The reason we use leave-one-out cross-validation is that we have a small data set and, moreover, there are only a small number of positive samples (hot spot residues). To test the robustness of our method, we evaluate performance using leave-*n*-out cross-validation under the same training-testing procedure. We find that when *n* is not large (< 7), the performance change is not significant, sometimes better (F1=0.6304, *n*=5) and sometimes worse (F1=0.5934. *n*=3) than that by using leave-one-out. Anyway, as shown later, no matter how large *n* is, our performance is always better than Robetta and FoldX. For example, the performance of our method by 5-fold (leave-51-out) cross-validation is 0.5870 in F measure. This indicates that our method is robust.

We compare our method with three energy-based methods, Robetta [19,21], FoldX [20,50] and EGAD [22]. Robetta is an online service. It can be used to predict the ∆∆*G* value of interfacial residues by computational alanine scanning based on an energetic function. It can thus be applied to hot spot prediction. Actually, it is a widely recognized gold standard for benchmark comparison in the field. Its performance on our data set is shown in the forth row of Table 1. Our performance is remarkably better than that of Robetta in terms of both F1 and p-value.

FoldX is also available online. It is able to predict the change in both protein stability and affinity. Its energy function contains different sources of contributions such as van der Waals interactions, hydrogen bonds and even water-bridged interactions. We calculated the ∆∆*G* of mutations in our data set by using FoldX version 3.0 beta 4 with default parameters. From the fifth row of Table 1, although FoldX shows a better performance than Robetta, probably due to the fact that it is being updated, our method still achieves a better performance than that by FoldX.

EDAG contains a group of energy functions for protein design and it has a module to predict the change in binding free energy after a mutation. We compare our method with EGAD based on the data that is common between our data set and those reported in their paper [22], namely 166 mutations (34 hot spot residues and 132 non hot spot residues). As shown in Table 2, our method’s performance is significantly better than that of EGAD. In fact, EGAD tends to underestimate the ∆∆*G* values—especially for the barnase-barstar complex [PDB:1BRS] in which there are many hot spot residues—thus many hot spot residues are misclassified as non hot spot, resulting in poor sensitivity.

**Table 2:** Comparison of our method with EGAD

Method	Sensitivity	Precision	Specificity	Accuracy	F1	p-value
DBAC	0.5294	0.6923	0.9394	0.8554	0.6	3.1412×10^–8^
EGAD	0.3235	0.5	0.9167	0.7952	0.3929	1.3693×10^–4^

These energy-based method are complicated and time-consuming. The energy functions usually contain many terms that represent different kinds of energies. Both binding and folding of proteins can affect the binding free energy between two proteins. But binding and folding are very complicated processes whose details are difficult to capture. When a residue is mutated into alanine, the new structure of the mutated protein and the mutated protein complex must be predicted to get the values of all energy terms of the mutated structure, which is also very difficult. Thus the ∆∆*G* are hard to be accurately estimated even by these complicated energy functions. From Tables 1 and 2, the performance of these energy-based methods are not very good yet.

We also compare our method with another machine learning method, MINERVA [15], which uses SVM as well and is based on a larger feature set containing various aspects of information of target residue such as weighted atomic packing density, relative surface area burial, weighted hydrophobicity and so on.

MINERVA has good performance in terms of F1 value in comparison with other previous machine-learning methods. Because its source codes and software are not available, we compare the performance on data that are common between the MINERVA paper and our data set. This common data set contains 178 mutations with 36 hot spot residues and 142 non hot spot residues. It can be seen from Table 3 that MINERVA does not outperform our method in terms of F measure or p-value. Moreover, the reported performance of MINERVA may be biased upwards by an overfitting effect because MINERVA uses 12 features selected from a total of 54 basing on the whole data set. MINERVA had also been tested on an independent data set derived from BID [51], where the importance of a residue is labeled as “strong”, “intermediate”, “weak” or “insignificant”. In that test, MINERVA treated a residue as a hot spot residue only when its label is “strong”. We also test our model (trained on our data set, 258 mutations) on a subset of this independent data set containing 111 mutations whose PDB files have associated solvent information. The performance of our model in terms of F1 on this data set is 52%, which is same as that of MINERVA. Note that the label of a residue is not perfectly correlated to its ∆∆*G*, which is an inconsistency between the training data and the test data; however, it still can indicate the contribution of a residue to binding.

**Table 3:** Comparison of our method with MINERVA

Method	Sensitivity	Precision	Specificity	Accuracy	F1	p-value
DBAC	0.5833	0.7	0.9366	0.8652	0.6364	2.4011×10^–9^
MINERVA	0.5	0.6667	0.9366	0.8483	0.571	1.3731×10^–7^

### Feature analysis

We test the significance of the difference of the values of a feature in the hot spot and non hot spot residues. The p-values are reported in Table 4. It is clear that the DBAC features have very low p-values, indicating that their values are significantly different between the two classes. The p-value of *C*(*I*,≥ 2) is not as low as that of other DBAC features, because there are fewer residues that have salt bridges. The numbers of slightly buried atomic contacts are not as significant as those of the deeply buried ones. Thus our feature set can indeed reflect the contrast between hot spot residues and non hot spot residues, and the idea of excluding slightly buried and exposed atomic contacts and using only deeply buried atomic contacts is statistically reasonable.

**Table 4:** Statistical analysis on the features.

Feature p-value	RBL* 5.0897 × 10^-10^	C(I, 0) 0.2008	C(I, 1) 0.8204	*C(I, ≥ 2)* *0.0050*
Feature p-value	C(II, 0) 0.0013	C(II, 1) 0.4133	*C(II, 2)* *1.2419 × 10^-9^*	*C(II, ≥ 3)* *3.5031 × 10^-6^*

Feature p-value	C(III, 0) 0.0034	C(III,1) 0.0945	*C(III, 2)* *1.9061 × 10^-16^*	*C(III, ≥ 3)* *1.1621 × 10^-9^*

The features in the feature set DBAC are emphasized in italics

*:residue burial level

We show in Table 5 the performance of our method after one feature is removed under the same training-testing protocol described in Methods. It seems that the removal of residue burial level, *C*(*I*, ≥ 2), *C*(*II*, 2) or *C*(*II*, ≥ 3) has little impact on performance. A reason is that the features we are using are somehow correlated with each other. In general, when an interfacial residue is deeply buried with high residue burial level, it has several deeply buried atomic contacts with the other side. On the other hand, the removal of *C*(*III*, 2) or *C*(*III*, ≥ 3) reduces performance a lot. The reason is that there are often many Type-III atomic contacts in hot spot residues, as Type-III atomic contacts is not as specific as Types I or II. This suggests that we can further divide Type-III atomic contacts into subtypes. We have actually tried dividing Type-III contacts into other polar-polar contacts and hydrophobic contacts; and it turns out that the performance change is not much and hydrophobic contacts become the new dominant one. However, this does not mean residue burial level, *C*(*I*, ≥ 2), *C*(*II*, 2) and *C*(*II*, ≥ 3) do not contribute to the performance because all the six features have significantly different values in hot spot and non hot spot residues. In fact, we can achieve an F measure of 0.5 with only the residue burial level.

Another interesting observation from Table 5 is that the performance after the removal of *C*(*II*, 2) (or *C*(*III*, 2) ) is better than that after the removal of *C*(*II*, ≥ 3) (or *C*(*III*, ≥ 3)), although the difference between hot spot and non hot spot residues in *C*(*II*, 2) or *C*(*III*, 2) is more significant (as shown in Table 4) and the number of residues that have Type-II or Type-III atomic contacts at burial level ≥ 3 is lower (as shown in Figure 3). As we have already shown that the performance with feature set AC is worse than that with DBAC, the observation here further indicates that the atomic contact at deeper burial level (≥ 3) is more important in hot spot prediction. This again confirms that burial level plays a very important role.

**Table 5:** Performances of our method after one feature is removed.

Feature Removed	RBL	C(I, ≥ 2)	C(II, 2)	C(II, ≥ 3)	C(III, 2)	C(III, ≥ 3)
F1	0.6	0.6067	0.6237	0.6	0.4828	0.3947

#### Residue burial level

**Figure 2:**
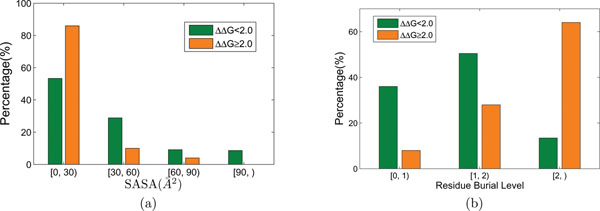
**Distribution of the SASA values (a), and distribution of the residue burial levels (b), in the hot spot and non hot spot residues**. The two figures here show a comparison between the distribution of the values of two residue descriptors: SASA that is usually used in hot spot prediction, and burial level that is proposed by us, in hot spot and non hot spot residues. In (a), the distribution of SASA is shown, in which we can find that both the number of hot spots and the number of non hot spots go down as SASA gets larger. In (b), as the burial level gets larger, the number of non hot spot residues drops while the number of hot spot residues goes up.

Residue burial level is a very important feature for predicting hot spot residues. Its p-value shows a very significant difference between hot spot residues and non hot spot residues, as can be seen in Table 4. Here, we explain that residue burial level is more sufficient than SASA in hot spot prediction. Bogan and Thorn [7] found that hot spot residues tend to have low SASA values. Based on this observation, they suggested the existence of a ring of energetically less important residues that are responsible of protecting the hot spot. Generally, a low SASA value is a necessary condition for a residue to become a hot spot residue. Thus, it is usually used someway for hot spot prediction. For example, the HotSprint database [13] defines computational hot spots as those conserved residues that have large SASA change (∆SASA) in complex formation and low SASA in the complex. However, a low SASA value is not a sufficient condition for a hot spot. As observed in Figure 2(a), in our data set, hot spot residues tend to have low SASA values with more than 80% of hot spot residues having SASA less than 30 Å^2^. But non hot spot residues also follow such a tendency (55%), albeit in a less remarkable yet observable way. We have tried to incorporate SASA into our model, by adding it to the feature set. The performance drops a lot, only 0.3188 by F measure.

In contrast, as shown in Figure 2(b), hot spot residues tend to have a high burial level, while non hot spot residues do not. More than 60% of hot spot residues have a burial level no less than 2.0, whereas less than 20% of non hot spot residues have such burial levels. Thus, we conjecture that a high burial level is not only necessary but also more sufficient than a low SASA value for a hot spot residue.

#### Deeply buried atomic contacts

Type-I atomic contacts roughly correspond to salt bridges. Some researchers believe that buried salt bridges provide neutral or even negative contribution to protein stability [52,53] because the desolvation of charged groups requires more energy than the interaction energy of the formation of the salt bridge [54]. But in protein-protein interaction, it is found that interfacial salt bridges are more buried than intra-chain salt bridges, and the salt bridges are found favorable across the interface [3]. Perhaps the two proteins are folded independently with more charged residues exposed and their conformation change during complex formation is very restricted, thus the two proteins prefer to interact in an electrostatic complementary manner.

**Figure 3:**
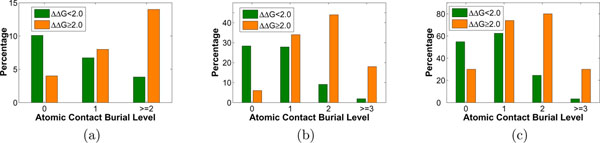
**Percentage of hot spot residues and non hot spot residues that have at least one Type-I (a), Type-II (b) or Type-III (c) directly broken interfacial atomic contact at different burial level**. The two figures here shows the percentage of hot spot residues and non hot spot residues that have at least one Type-I (a), Type-II (b) or Type-III (c) directly broken interfacial atomic contact at different burial level. It can be noted that the values for deeply buried atomic contacts are significantly different in hot spot and non hot spot residues.

Percentages of hot spot residues and non hot spot residues whose *C*(*I*, *x*) are larger than 0 are plotted in Figure 3(a). Generally, hot spot residues tend to have their salt bridges buried while non hot spot residues do not. Two adjacent exposed oppositely charged groups may not form stable salt bridges at all [4]; thus some exposed Type-I contacts, most of which are possessed by non hot spot residues, may not be stable salt bridges.

Hydrogen bonds play a key role in protein-protein interaction [2]. Most interfacial hydrogen bonds are extremely buried; and the more buried a hydrogen bond donor/acceptor is, the more likely it is to form a hydrogen bond [3]. Thus, being buried is favorable for interfacial hydrogen bonds. Figure 3(b) shows the percentages of hot spot residues and non hot spot residues whose *C*(*II*, *x*) are larger than 0. It can be seen that nearly 30% of non hot spot residues have exposed Type-II atomic contacts, but very few of them have deeply buried hydrogen bonds. The case is totally different in hot spot residues. There are more hot spot residues that have deeply buried Type-II atomic contact while a few of them have exposed ones. The number of residues that have extremely buried (burial level≥ 3) atomic contacts is limited by the size of the protein complexes.

Type-III contacts contain all other kinds of contacts that are neither salt bridges nor hydrogen bonds, including hydrophobic contacts and other polar contacts. Actually hydrophobic contacts are not specific contact between atoms but are the packing of groups of hydrophobic side chains. The contribution of hydrophobic contacts to bonding free energy is correlated with the buried surface area [6]. Thus energetically important hydrophobic contacts are those buried ones. Generally, protein-protein interfaces are dominated by salt bridges, hydrogen bonds and hydrophobic contacts; but sometimes other contacts also make contribution to the binding [3]. A hot spot is usually a densely packed region in the interface, thus the number of buried contacts of a hot spot residue tends to be large, which can be reflected by deeply buried Type-III contacts. As shown in Figure 3(c), more than 80% of hot spot residues have Type-III contact at burial level 2 and only about 20% non hot spot residues have Type-III contact at this burial level.

### Case study: three residues that are difficult to classify

**Figure 4:**
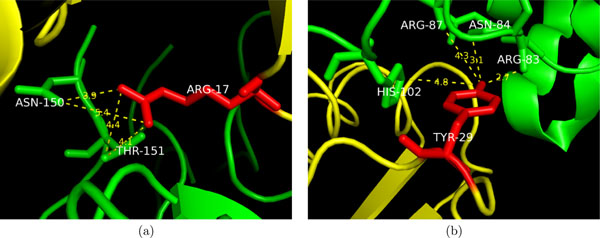
**Two residues that are difficult to classify in our data set.** Two residues that are difficult to classify in our data set. In (a), ARG-17 of BPTI (in yellow) in bovine chymotrypsin (in green)-BPTI complex [PDB:1CBW] is shown. This residue is a non hot spot residue (∆∆G=0.5 kcal/mol) with an extremely low SASA (8.0 Å^2^). In (b), TYR-29 of barstar (in yellow) in barnase (in green)-barstar complex [PDB:1BRS] is shown. This residue is a hot spot residue (∆∆G=3.4 kcal/mol), but it has a large SASA (64.12 Å^2^). These two residues have been correctly classified by our method. The dashed lines in the figures are Type-II atomic contacts (candidates of hydrogen bonds).

Figure 4 shows the structure of two residues that are difficult to classify. ARG-17 of BPTI shown in Figure 4(a) is well buried in the interface of the complex with a very low SASA of 8.0Å^2^, a small SASA value that is not even enough to define an exposed atom. Arginines are actually very likely to be hot spot residues [7,9], especially when they have such a low SASA. However, this ARG-17 is a non hot spot residue, having a ∆∆*G* of only 0.5 kcal/mol. Its burial level is 1.55, which is not a high value and, more importantly, almost all its atomic contacts with bovine chymotrypsin are just slightly buried or even exposed. There are 4 Type-II contacts shown in the figure, with 2 exposed and 2 slightly buried. It also has another 15 Type-III contacts, with 13 slightly buried, 1 exposed and 1 deeply buried. We successfully classified this residue as a non hot spot residue.

Another example as shown in Figure 4(b) is TYR-29 of barstar. This residue is exposed with an SASA of 64.12 Å^2^; however, it is a hot spot residue with a ∆∆*G* of 3.4 kcal/mol. There are only two hot spot residues that have SASA larger than 60Å^2^ in our data set. We can still successfully identify it as a hot spot residue by using its deeply buried atomic contacts. The side-chain of tyrosine which contains an aromatic ring and a hydroxyl group is capable of forming aromatic *π*-interactions and hydrogen bonds [7]. As can be seen from the figure, although TYR-29 of barstar is partially exposed, its side-chain stretches into the complex and forms many deeply buried atomic contacts. For the 4 Type-II interfacial atomic contacts shown in the figure, 3 are deeply buried and 1 is slightly buried. There are another 8 deeply buried Type-III contacts, 7 of which are made by the aromatic ring and 5 are atomic contacts with HIS-102, an active site residue of barnase [55].

**Figure 5:**
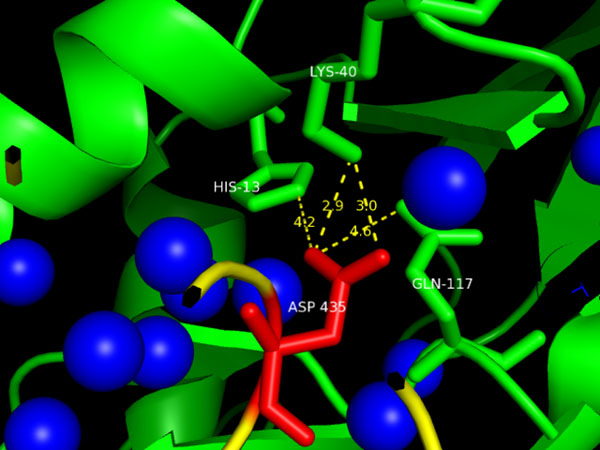
**A case that only our method works.** ASP-435, a residue of ribonuclease inhibitor (in yellow) in ribonuclease inhibitor-angiogenin (in green) complex [PDB:1A4Y] that we have successfully classified as a hot spot residue while Robetta, FoldX, EGAD and MINERVA failed. This residue has several nearby water molecules (in blue spheres), which may be the reason why other methods cannot predict its type successfully. These water molecules are buried water molecules are considered as part of the protein complex by our method.

In Figure 5, we show another residue—ASP-435 of ribonuclease inhibitor in ribonuclease inhibitor-angiogenin complex—that is in a “wet” local environment, although it is buried inside the interface and well wrapped. It is a hot spot residue with a ∆∆*G* of 3.5 kcal/mol. As can be seen from the figure, this residue has several nearby water molecules, which may be the reason Robetta, FoldX, EGAD and MINERVA have failed to classify it as a hot spot residue. We consider buried water molecules as part of the protein complex; thus, the buried water molecules shown in the figure not only are buried but also are shelters of the nearby residues. This residue has 3 Type-I, 1 Type-II and 11 Type-III deeply buried atomic contacts. If we do not consider buried water molecules as part of the complex, these atomic contacts will no longer be deeply buried.

## Conclusions

We have proposed a feature-based method to predict protein-binding hot spots by using deeply buried interfacial atomic contacts that are directly broken during alanine substitution. The method is based on a graph theoretical definition of burial level of residues, atoms and atomic contacts. We achieved an F measure of 0.6237 when ∆∆*G* ≥ 2.0 is used as the threshold to define hot spot residues. The burial level of a residue is more intuitive than the concept of SASA. It is nicely correlated with the ∆∆*G* of a residue. We have shown that a high residue burial level is in general necessary for a residue to be a hot spot residue. Furthermore, it is more sufficient than SASA, a frequently used feature in existing hot spot prediction methods. Our results also reveal that hot spot residues tend to have deeply buried atomic contacts while non hot spots tend to have exposed and slightly buried ones. This is consistent with previous studies that emphasize the energetic contribution of buried salt bridges, hydrogen bonds and hydrophobic contacts.

## Competing interests

The authors declare that there are no competing interests.

## Authors’ contributions

ZL, JL and LW discussed and designed the experiment; ZL conducted the experiment and wrote the paper; JL and LW revised the paper. All authors read and approved the final manuscript.

## References

[B1] Jones S Thornton JM Principles of protein-protein interactions Proc Natl Acad Sci U S A 1996 93 13 20 10.1073/pnas.93.1.13 8552589 PMC40170

[B2] Keskin O Gursoy A Ma B Nussinov R Principles of protein-protein interactions: what are the preferred ways for proteins to interact? Chem Rev 2008 108 4 1225 1244 10.1021/cr040409x 18355092

[B3] Xu D Tsai CJ Nussinov R Hydrogen bonds and salt bridges across protein-protein interfaces Protein Eng 1997 10 9 999 1012 10.1093/protein/10.9.999 9464564

[B4] Sun DP Sauer U Nicholson H Matthews BW Contributions of engineered surface salt bridges to the stability of T4 lysozyme determined by directed mutagenesis Biochemistry 1991 30 29 7142 53 10.1021/bi00243a015 1854726

[B5] Fernàndez A Scheraga HA Insufficiently dehydrated hydrogen bonds as determinants of protein interactions Proc Natl Acad Sci U S A 2003 100 113 118 10.1073/pnas.0136888100 12518060 PMC140898

[B6] Sharp KA Nicholls A Fine RF Honig B Reconciling the magnitude of the microscopic and macroscopic hydrophobic effects Science 1991 252 5002 106 109 10.1126/science.2011744 2011744

[B7] Bogan AA Thorn KS Anatomy of hot spots in protein interfaces J Mol Biol 1998 280 1 9 10.1006/jmbi.1998.1843 9653027

[B8] Keskin O Ma B Nussinov R Hot regions in protein-protein interactions: the organization and contribution of structurally conserved hot spot residues J Mol Biol 2005 345 5 1281 1294 10.1016/j.jmb.2004.10.077 15644221

[B9] Moreira ISS Fernandes PAA Ramos MJJ Hot spots-A review of the protein-protein interface determinant amino-acid residues Proteins 2007 68 4 803 812 10.1002/prot.21396 17546660

[B10] Cunningham B Wells J High-resolution epitope mapping of hGH-receptor interactions by alanine-scanning mutagenesis Science 1989 244 4908 1081 1085 10.1126/science.2471267 2471267

[B11] Wells JA Langone JJ Systematic mutational analyses of protein-protein interfaces Molecular Design and Modeling: Concepts and Applications Part A: Proteins, Peptides, and Enzymes, Volume 202 of Methods in Enzymology 1991 Academic Press 390 411 full_text 1784171

[B12] Clackson T Wells JA A hot spot of binding energy in a hormone-receptor interface Science 1995 267 5196 383 386 10.1126/science.7529940 7529940

[B13] Guney E Tuncbag N Keskin O Gursoy A HotSprint: database of computational hot spots in protein interfaces Nucleic Acids Res 2007 36 Database D662 D666 10.1093/nar/gkm813 17959648 PMC2238999

[B14] Darnell SJ Page D Mitchell JC An automated decision-tree approach to predicting protein interaction hot spots Proteins 2007 68 4 813 823 10.1002/prot.21474 17554779

[B15] Cho KI Kim D Lee D A feature-based approach to modeling protein-protein interaction hot spots Nucleic Acids Res 2009 37 8 2672 2687 10.1093/nar/gkp132 19273533 PMC2677884

[B16] Tuncbag N Gursoy A Keskin O Identification of computational hot spots in protein interfaces: combining solvent accessibility and inter-residue potentials improves the accuracy Bioinformatics 2009 25 12 1513 1520 10.1093/bioinformatics/btp240 19357097

[B17] Xia JF Zhao XM Song J Huang DS APIS: accurate prediction of hot spots in protein interfaces by combining protrusion index with solvent accessibility BMC Bioinformatics 2010 11 174 10.1186/1471-2105-11-174 20377884 PMC2874803

[B18] Massova I Kollman PA Computational Alanine Scanning To Probe Protein-Protein Interactions: A Novel Approach To Evaluate Binding Free Energies J Am Chem Soc 1999 121 36 8133 8143 10.1021/ja990935j

[B19] Kortemme T Baker D A simple physical model for binding energy hot spots in protein-protein complexes Proc Natl Acad Sci U S A 2002 99 22 14116 14121 10.1073/pnas.202485799 12381794 PMC137846

[B20] Guerois R Nielsen JE Serrano L Predicting Changes in the Stability of Proteins and Protein Complexes: A Study of More Than 1000 Mutations J Mol Biol 2002 320 2 369 387 10.1016/S0022-2836(02)00442-4 12079393

[B21] Kortemme T Kim DE Baker D Computational Alanine Scanning of Protein-Protein Interfaces Sci STKE 2004 2004 219 pl2 10.1126/stke.2192004pl2 14872095

[B22] Pokala N Handel TM Energy Functions for Protein Design: Adjustment with Protein-Protein Complex Affinities, Models for the Unfolded State, and Negative Design of Solubility and Specificity J Mol Biol 2005 347 203 227 10.1016/j.jmb.2004.12.019 15733929

[B23] Benedix A Becker CM de Groot BL Caflisch A Bockmann RA Predicting free energy changes using structural ensembles Nature Methods 2009 6 3 4 10.1038/nmeth0109-3 19116609

[B24] Lise S Archambeau C Pontil M Jones D Prediction of hot spot residues at protein-protein interfaces by combining machine learning and energy-based methods BMC Bioinformatics 2009 10 365 10.1186/1471-2105-10-365 19878545 PMC2777894

[B25] Assi SA Tanaka T Rabbitts TH Fernandez-Fuentes N PCRPi: Presaging Critical Residues in Protein interfaces, a new computational tool to chart hot spots in protein interfaces Nucleic Acids Res 2010 38 6 10.1093/nar/gkp1158 20008102 PMC2847225

[B26] Thorn KS Bogan AA ASEdb: a database of alanine mutations and their effects on the free energy of binding in protein interactions Bioinformatics 2001 17 3 284 285 10.1093/bioinformatics/17.3.284 11294795

[B27] Cunningham BC Wells JA Comparison of a Structural and a Functional Epitope J Mol Biol 1993 234 3 554 563 10.1006/jmbi.1993.1611 7504735

[B28] Dougan D Malby R Gruen L Kortt A Hudson P Effects of substitutions in the binding surface of an antibody on antigen affinity Protein Eng 1998 11 65 74 10.1093/protein/11.1.65 9579662

[B29] Clackson T Ultsch MH Wells JA de Vos AM Structural and Functional Analysis of the 1:1 Growth Hormone:Receptor Complex Reveals the Molecular Basis for Receptor Affinity J Mol Biol 1998 277 5 1111 1128 10.1006/jmbi.1998.1669 9571026

[B30] Pineda AO Cantwell AM Bush LA Rose T Di Cera E The thrombin epitope recognizing thrombomodulin is a highly cooperative hot spot in exosite I J Biol Chem 2002 277 35 32015 32019 10.1074/jbc.M205009200 12068020

[B31] Reichmann D Rahat O Albeck S Meged R Dym O Schreiber G The modular architecture of protein-protein binding interfaces Proc Natl Acad Sci U S A 2005 102 57 62 10.1073/pnas.0407280102 15618400 PMC544062

[B32] Berman HM Westbrook J Feng Z Gilliland G Bhat TN Weissig H Shindyalov IN Bourne PE The Protein Data Bank Nucleic Acids Res 2000 28 235 242 10.1093/nar/28.1.235 10592235 PMC102472

[B33] Shindyalov IN Bourne PE Protein structure alignment by incremental combinatorial extension (CE) of the optimal path Protein Eng 1998 11 9 739 747 10.1093/protein/11.9.739 9796821

[B34] Li Z Li J Geometrically centered region: A “wet” model of protein binding hot spots not excluding water molecules Proteins 2010 78 16 3304 3316 10.1002/prot.22838 20818601

[B35] Lee B Richards FM The interpretation of protein structures: estimation of static accessibility J Mol Biol 1971 55 3 379 400 10.1016/0022-2836(71)90324-X 5551392

[B36] Barber BC Dobkin DP Huhdanpaa H The Quickhull Algorithm for Convex Hulls ACM Transactions on Mathematical Software 1996 22 4 469 483 10.1145/235815.235821

[B37] Li J Liu Q’Double water exclusion’: a hypothesis refining the O-ring theory for the hot spots at protein interfaces Bioinformatics 2009 25 6 743 750 10.1093/bioinformatics/btp058 19179356 PMC2654803

[B38] Dijkstra EW A note on two problems in connexion with graphs Numerische Mathematik 1959 1 269 271 10.1007/BF01386390

[B39] Chakravarty S Varadarajan R Residue depth: a novel parameter for the analysis of protein structure and stability Structure 1999 7 7 723 732 10.1016/S0969-2126(99)80097-5 10425675

[B40] Pintar A Carugo O Pongor S DPX: for the analysis of the protein core Bioinformatics 2003 19 2 313 314 10.1093/bioinformatics/19.2.313 12538266

[B41] Pintar A Atom Depth as a Descriptor of the Protein Interior Biophysical Journal 2003 84 4 2553 2561 10.1016/S0006-3495(03)75060-7 12668463 PMC1302821

[B42] Pintar A Carugo O Pongor S Atom depth in protein structure and function Trends Biochem Sci 2003 28 11 593 597 10.1016/j.tibs.2003.09.004 14607089

[B43] Hamelryck T An amino acid has two sides: a new 2D measure provides a different view of solvent exposure Proteins 2005 59 38 48 10.1002/prot.20379 15688434

[B44] Song J Tan H Mahmood K Law RHP Buckle AM Webb GI Akutsu T Whisstock JC Prodepth: Predict Residue Depth by Support Vector Regression Approach from Protein Sequences Only PLoS ONE 2009 4 9 e7072 10.1371/journal.pone.0007072 19759917 PMC2742725

[B45] Yuan Z Wang ZX Quantifying the relationship of protein burying depth and sequence Proteins: Structure Function, and Bioinformatics 2008 70 2 509 516 10.1002/prot.21545 17705271

[B46] Zhang H Zhang T Chen K Shen S Ruan J Kurgan L Sequence based residue depth prediction using evolutionary information and predicted secondary structure BMC Bioinformatics 2008 9 388 10.1186/1471-2105-9-388 18803867 PMC2567998

[B47] Pace CN Grimsley GR Scholtz JM Protein Ionizable Groups: pK Values and Their Contribution to Protein Stability and Solubility J Biol Chem 2009 284 20 13285 13289 10.1074/jbc.R800080200 19164280 PMC2679426

[B48] Chang CC Lin CJ LIBSVM: a library for support vector machines 2001 http://www.csie.ntu.edu.tw/~cjlin/libsvm

[B49] Mann HB Whitney DR On a Test of Whether one of Two Random Variables is Stochastically Larger than the Other The Annals of Mathematical Statistics 1947 18 50 60 10.1214/aoms/1177730491

[B50] Schymkowitz J Borg J Stricher F Nys R Rousseau F Serrano L The FoldX web server: an online force field Nucleic Acids Res 2005 33 10.1093/nar/gki387 PMC1160148 15980494

[B51] Fischer TB Arunachalam KV Bailey D Mangual V Bakhru S Russo R Huang D Paczkowski M Lalchandani V Ramachandra C Ellison B Galer S Shapley J Fuentes E Tsai J The binding interface database (BID): a compilation of amino acid hot spots in protein interfaces Bioinformatics 2003 19 11 1453 1454 10.1093/bioinformatics/btg163 12874065

[B52] Albeck S Unger R Schreiber G Evaluation of direct and cooperative contributions towards the strength of buried hydrogen bonds and salt bridges J Mol Biol 2000 298 3 503 520 10.1006/jmbi.2000.3656 10772866

[B53] Waldburger CD Schildbach JF Sauer RT Are buried salt bridges important for protein stability and conformational specificity? Nat Struct Biol 1995 2 122 128 10.1038/nsb0295-122 7749916

[B54] Hendsch ZS Tidor B Do salt bridges stabilize proteins? A continuum electrostatic analysis Protein Sci 1994 3 2 211 226 10.1002/pro.5560030206 8003958 PMC2142793

[B55] Buckle AM Schreiber G Fersht AR Protein-protein recognition: Crystal structural analysis of a barnase-barstar complex at 2.0Å resolution Biochemistry 1994 33 30 8878 8889 10.1021/bi00196a004 8043575

